# Aberrant ASPM expression mediated by transcriptional regulation of FoxM1 promotes the progression of gliomas

**DOI:** 10.1111/jcmm.15435

**Published:** 2020-07-15

**Authors:** Wen‐Jing Zeng, Quan Cheng, Zhi‐Peng Wen, Jie‐Ya Wang, Yan‐Hong Chen, Jie Zhao, Zhi‐Cheng Gong, Xiao‐Ping Chen

**Affiliations:** ^1^ Department of Clinical Pharmacology Xiangya Hospital Central South University Changsha China; ^2^ Hunan Key Laboratory of Pharmacogenetics Institute of Clinical Pharmacology Central South University Changsha China; ^3^ Department of Pharmacy Xiangya Hospital Central South University Changsha China; ^4^ Neurosurgery Xiangya Hospital Central South University Changsha China; ^5^ National Clinical Research Center for Geriatric Disorders (XIANGYA) Xiangya Hospital Central South University Changsha China

**Keywords:** ASPM, biomarker, FoxM1, glioma

## Abstract

Gliomas are the most common form of malignant tumour in the central nervous system. However, the molecular mechanism of the tumorigenesis and progression of gliomas remains unclear. In this study, we used the GEO database to identify genes differentially expressed in gliomas and predict the prognosis of glioma. We observed that *ASPM* mRNA was increased obviously in glioma tissue, and higher *ASPM* mRNA expression predicted worse disease prognosis. ASPM was highly expressed in glioma cell lines U87‐MG and U251, and knockdown of ASPM expression in these cells significantly repressed the proliferation, migration and invasion ability and induced G0/G1 phase arrest. In addition, down‐regulation of ASPM suppressed the growth of glioma in nude mice. Five potential binding sites for transcription factor FoxM1 were predicted in the *ASPM* promoter. FoxM1 overexpression significantly increased the expression of ASPM and promoted the proliferation and migration of glioma cells, which was abolished by ASPM ablation. ChIP and dual‐luciferase reporter analysis confirmed that FoxM1 bound to the *ASPM* promoter at −236 to ‐230 bp and −1354 to ‐1348 bp and activated the transcription of ASPM directly. Collectively, our results demonstrated for the first time that aberrant ASPM expression mediated by transcriptional regulation of FoxM1 promotes the malignant properties of glioma cells.

## 1 INTRODUCTION

Gliomas are the most common primary malignant tumour of the central nervous system in adults, accounting for 27% of all primary brain tumours and 80% of primary malignant brain tumours.^1^, ^2^ The histopathological characteristics and prognosis of gliomas show significant individual difference. Currently, for first‐line treatment of glioblastomas, the NCCN guidelines recommend maximal safe resection with or without radiation therapy and chemotherapy.^3^, ^4^ Due to the aggressive and invasive nature of malignant glioma, complete neurosurgical resection is impractical and the residual tumour cells are prone to resistant to radiotherapy and chemotherapy, resulting in poor prognosis and high recurrence of glioma patients. Therefore, further clarification of the molecular mechanism of the pathogenesis and progression of gliomas is warranted for the development new therapeutic targets for the disease.

*ASPM* (*abnormal spindle‐like microcephaly*) gene, also known as *MCPH5*, is the most common family microcephaly mutation gene involved in the regulation of neurogenesis and cerebral cortical size.^5^, ^6^ Intracellular ASPM is mainly distributed in centrosome and spindle microtubule intermediates, and ASPM deficiency leads to spindle assembly and mitotic process disruption.^7^, ^8^ In addition, ASPM is also implicated in tumorigenesis and tumour progression.^9^, ^10^, ^11^ In gliomas, the expression of ASPM increased with the glioma grade, and ASPM expression is significantly higher in recurrent glioma than that in primary gliomas.^12^ Depletion of ASPM suppresses the proliferation of glioma spheres and promotes cell death.^13^ Yet, the underlying molecular mechanisms that mediate the up‐regulation of ASPM expression in glioma tissues remain unknown.

FoxM1 is a member of the forkhead box (Fox) transcription factor family.^14^ Previous evidence indicated that FoxM1 plays important roles in a variety of biological processes including mitotic G1/S and G2/M phase transition, mitotic spindle integrity, DNA damage repair, angiogenesis and tumour metastasis.^14^, ^15^, ^16^ FoxM1 is highly expressed in a variety of solid tumours and contributes to malignant transformation and chemotherapy resistance, suggesting that FoxM1 may serve as a potential antitumour target.^17^, ^18^ FoxM1 is also highly expressed in glioblastoma (GBM), and higher FoxM1 expression is associated with worse overall survival in glioma patients. Mechanism studies have indicated that FoxM1 can promote glioma progression by enhancing MMP2 transcription^19^ or temozolomide (TMZ) resistance by up‐regulating RFC5 expression directly.^20^ In an attempt to find potential transcript factors that may play a role in increased ASPM expression in gliomas, we predicted several potential FoxM1 binding sites in ASPM promoter using transcript factor binding site prediction databases. However, whether FoxM1 can directly regulate ASPM expression and whether the FoxM1‐ASPM axis contributes to the pathogenesis and progression of gliomas remain largely unclear.

In this study, we aimed to identify genes related to the pathogenesis and progression and prognosis of gliomas based on GEO data sets and investigate the effects of ASPM on the biological functions of glioma cells, as well as the role of FoxM1 in the up‐regulation of ASPM expression in gliomas. Our results demonstrated for the first time that FoxM1 can directly activate the expression of ASPM by binding to the promoter, thereby regulating the proliferation, migration and invasion of glioma cells.

## 2 MATERIALS AND METHODS

### 2.1 Patient samples

All glioma and normal brain control tissue samples were obtained from patients undergoing neurosurgical resection in Xiangya Hospital of Central South University (Changsha, Hunan, China) between June 2015 and December 2017. All samples were confirmed by post‐operative pathology and classified according to the WHO Classification of the Central Nervous System Tumors. All procedures were approved by the Ethics Committee of Xiangya Hospital of Central South University, and informed consent was obtained from patients.

### 2.2 Bioinformatic data mining

GSE4271, GSE4290 and GSE45921 were downloaded from the Gene Expression Omnibus database, http://www.ncbi.nlm.nih.gov/geo/. 100 glioma samples from GSE4271, 157 gliomas samples and 23 non‐tumour samples from GSE4290 and 21 glioma samples from GSE45921 were used to screen genes differentially expressed in non‐tumour brain tissue and glioma tissue. Genes with |log fold change| ≥ 1 and adjusted *P* < .05 were considered as differentially expressed genes (DEGs).^21^ GO functional enrichment analysis and KEGG pathway enrichment analysis were performed on the DEGs using the DAVID online database. The CGGA and TCGA glioma data sets were used for prognostic analysis of DEGs. CGGA expression profile data and clinical information with 225 samples (5 non‐tumour brain tissues from epileptic patients and 220 glioma tissues) were downloaded at http://cgga.org.cn/. TCGA‐LGG and TCGA‐GBM mRNA expression data and related clinical information were downloaded at https://genome‐cancer.ucsc.edu/ (version 2015‐02‐24). 473 LGG patients and 539 patients GBM with complete clinical information were included in the TCGA‐LGG data set and TCGA‐GBM data set, respectively.

### 2.3 Cell culture and siRNA transfection

Human glioma cell lines U251‐MG and U87‐MG were obtained from National Infrastructure of Cell Line Resource of China. Human glioma cell lines Hs683, A172, T98G and U343 were kindly provided by Dr Lv, Jiangxi Cancer Hospital, China. Cells were cultured in DMEM (Gibco, CA, USA) supplemented with 10% (v/v) foetal bovine serum (Gibco, CA, USA) at 37°C in a humidified incubator with 5% CO_2_. U251‐MG or U87‐MG cells were seeded in 6‐well plates at a density of 2 × 10^5^ cells/well. After 24‐hour incubation, cells were transfected with different siRNAs using RNAiMAX according to the manufacturer's instructions.

### 2.4 RNA isolation and quantitative real‐time PCR

Total RNA was extracted from glioma tissues or cultured cells using RNAiso Plus reagent (Takara, Beijing, China), and then, cDNA synthesis was carried out with PrimeScript™ RT Reagent Kit (Takara, Beijing, China) according to the manufacturer's directions. The mRNA expression level of the target gene was detected using the fluorescence quantitative Real Time PCR Kit (Takara, Beijing, China) with the Roche LightCycler^®^ 480 System. Real‐time PCR primers for genes are shown in Table S2.

### 2.5 Immunohistochemical staining

Paraffin‐embedded glioma tissue sections were dewaxed with xylene and then hydrated with gradient ethanol. After antigen repair and blocking, the tissue sections were incubated with ASPM (Santa Cruz, CA, USA)‐ and FoxM1 (GeneTex, CA, USA)‐specific primary antibodies in a wet box at 4°C overnight. Negative control sections were in parallel incubated with non‐specific IgG (Sigma, MO, USA). Then, tissue sections were incubated with a secondary antibody for 2 hours at room temperature. After staining with DAB and haematoxylin, tissue sections were observed and photographed under a microscope.

### 2.6 Western blot analysis

The treated cells were collected and lysed with RIPA lysis buffer (Beyotime, Shanghai, China). The protein concentration was determined using BCA Protein Assay Kit (Beyotime, Shanghai, China). Equal amounts of proteins were separated by SDS‐PAGE and transferred to PVDF membranes (Millipore, MA, USA). After blocking, the membranes were immunoblotted with primary antibodies that were specific for ASPM, CyclinD1 and CyclinE (Santa Cruz, CA, USA); FoxM1 (GeneTex, CA, USA); P53, P21 and N‐cadherin (Cell Signaling Technology, MA, USA); GAPDH (Proteintech, Wuhan, China); and E‐cadherin, Vimentin, MMP1, MMP2, MMP9, RB and CDK2 (Wanlei Biotech, Shenyang, China). After washing with TBST, the membranes were incubated with appropriate secondary antibodies. The membranes were then exposed to enhanced Chemiluminescence‐Plus reagents (Beyotime, Shanghai, China). ChemiDoc™ XRS + chemiluminescence imaging system was used to capture the images. GAPDH protein was used as the internal reference to calculate the relative expression level of the protein by Image Lab 3.0 software.

### 2.7 Cell proliferation detection

Cell viability and proliferation were measured by CCK8 assay according to the manufacturer's instructions. In brief, cells were seeded in 96‐well plates at a density of 1 × 10^3^ cells/well. At the indicated time‐point, 90 μL culture medium and 10 μL CCK8 were added into each well. After gently mixing, cells were incubated at 37°C for 1 hour. Subsequently, the absorbance at 450 nm was detected by a microplate reader (Bio‐Rad, CA, USA), and the proliferation rate of glioma cells was calculated. Clonal formation assay was used to determine the proliferation ability of glioma cells, and the procedures were carried out according to a previous study.^22^

### 2.8 Wound healing assay

Wound healing assay was performed according to the previous study.^22^ In brief, cells were seeded in 6‐well plates at a density of 5 × 10^5^ cells/wells and cultured for 24 hours. After transfection for 24 hours, wounds were gently made using a micropipette tip, and the cells were washed with sterile PBS solution to remove floating cells. Then, the cells were cultured with serum‐free medium for 24 hours. Cells were photographed at 0 hour and 24 hours after scratch under an inverted microscope.

### 2.9 Transwell migration assay

The treated glioma cells were collected and resuspended in serum‐free medium at a final density of 5 × 10^5^ cells/well. 200 μL of cell suspension was added to the upper chamber of transwell, and 600 μL culture medium containing 15% FBS was added to the lower chamber. After incubation for 24 hours, the medium was removed, and the chamber was washed with PBS. Then, cells were fixed with 600 μL methanol for 15 minutes. Methanol was removed, and the cells in the upper chamber were gently wiped with a cotton swab. Subsequently, the cells on the underside of the membrane were stained with 0.1% crystal violet solution for 5 minutes. After washing with PBS, cells were observed and photographed under an inverted microscope.

### 2.10 Cell cycle analysis

The procedure was according to a previous study,^23^, ^24^ and the distribution of cell cycle was determined by flow cytometry (Beckman Coulter, CA, USA).

### 2.11 Dual‐luciferase reporter assay

Human FoxM1‐overexpressed plasmid and control plasmid were obtained from GeneChem (Shanghai, China). Human ASPM promoter reporter plasmids and pGL3‐Basic vector were purchased from BoChu Biotechnology (Changsha, China). PRL‐TK vector was kindly provided by Dr Cui, Central South University, China. Glioma cells were transfected with luciferase reporter plasmid and lysed 24 hours later. Subsequently, the firefly luciferase activity and Renilla luciferase activity were determined with the dual‐luciferase assay system (Promega, WI, USA) according to the manufacturer's directions.

### 2.12 Chromatin immunoprecipitation (ChIP) PCR assay

ChIP experiments were performed with the commercially available Pierce™ Agarose ChIP Kit (No. 26156) (Thermo Fisher, CA, USA) according to the manufacturer's instructions. Real‐time PCR primers for the ASPM promoter region are shown in Table S3.

### 2.13 Subcutaneous tumorigenesis experiment of nude mice

Female BALB/c nude mice aged 6 weeks and weighing about 18 g were selected for this experiment. Sixteen mice were randomly divided into sh‐con group and sh‐ASPM group. ASPM stable knockout U87‐MG cells (U87‐MG‐sh‐ASPM cells) and knockdown control U87‐MG cells (U87‐MG‐sh‐con cells) were collected and resuspended in pre‐cooled DMEM containing 10% Matrigel and 2% FBS at a final density of 5 × 10^7^ cells/mL. Then, mice were injected subcutaneously with 200 μL cell suspension. After tumour implantation, animal reactions were observed. Nude mice were weighed every 2 days, and the long and short diameters of tumours were measured. The tumour volume was calculated as previously described.^25^ After 21 days of transplantation, the mice were killed and tumour tissues were completely dissected. All of the procedures were conducted according to the guidelines of the Institutional Animal Care and Use Committee of Central South University and were approved by the Institutional Ethics Committee of Central South University.

### 2.14 Statistical analysis

All of the statistical analyses were carried out using SPSS 19.0 software (SPSS Inc, IL, USA) and GraphPad Prism 5.0 (GraphPad Inc, CA, USA). The data are presented as mean ± SD. Student's *t* test was used to analyse the significance of the differences between groups, and one‐way ANOVA was used to analyse significance of the differences among groups. Kaplan‐Meier survival analysis was used to determine the survival profiles, and log‐rank test was carried out to assess the statistical significance of differences. *P* < .05 was considered statistically significant.

## 3 RESULTS

### 3.1 ASPM is related to glioma risk and prognosis

**FIGURE 1:**
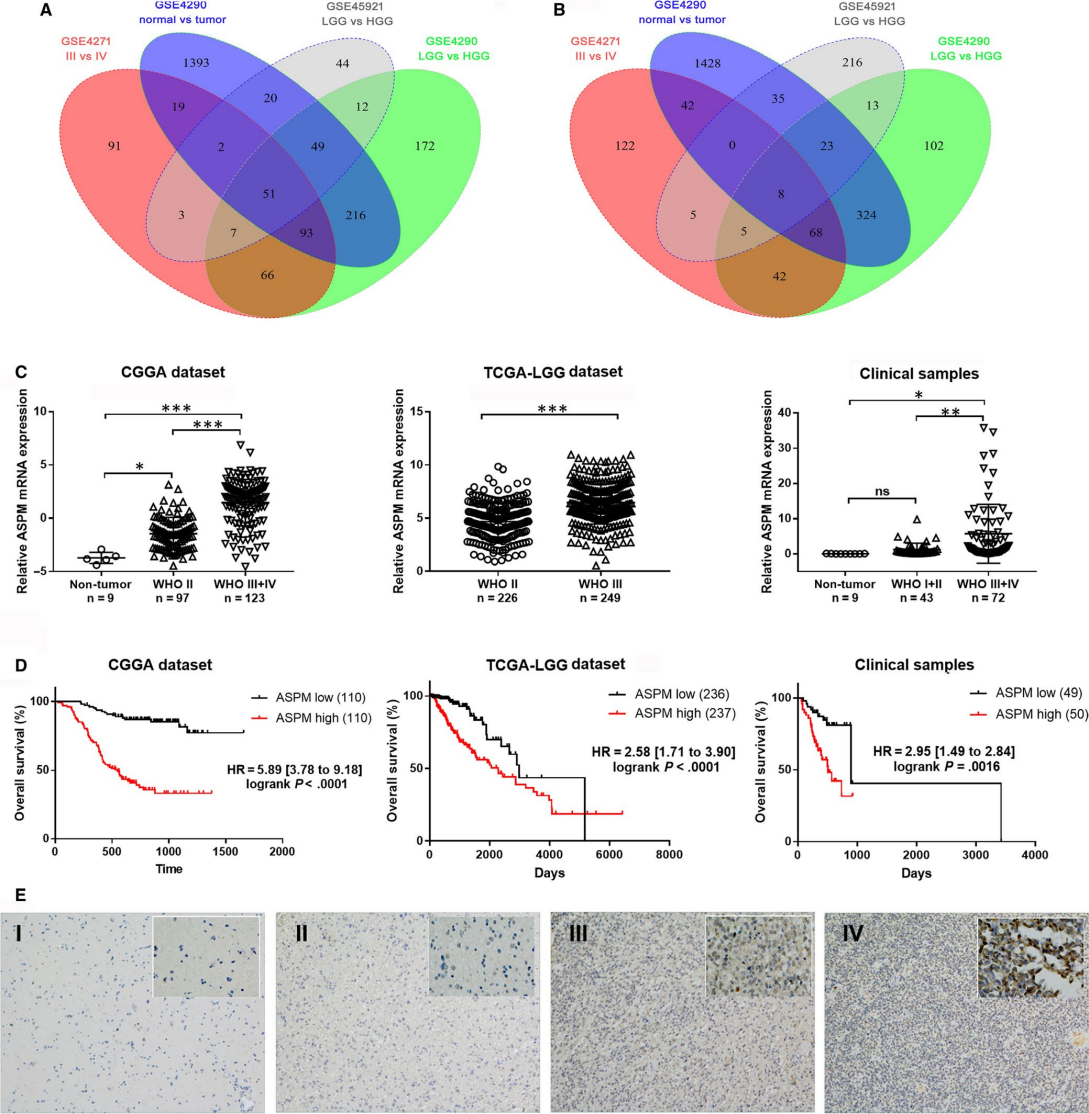
ASPM is related to glioma risk and prognosis. A, B, Venn diagram showing DEGs in the data set of GSE4290, GSE4271 and GSE45921. C, The expression of *ASPM* mRNA in normal brain tissues and glioma tissues at different pathological grade in CGGA data set, TCGA‐LGG data set and clinical samples; *P < 0.05, **P < 0.01, ***P < 0.001. D, Kaplan‐Meier plot for overall survival between patients with high and low expression of *ASPM* mRNA in CGGA data set, TCGA‐LGG data set and clinical samples. E, IHC detection of ASPM protein expression in glioma tissues. The sections were photographed at 100× and 400× magnification

Three gene expression profiles (GSE4290, GSE4271 and GSE45921) were used to identify genes related to the pathogenesis and progression of gliomas. As shown in Figure 1A,B and Table 1, a total of 59 differentially expressed genes (DEGs) were found between normal brain tissues and glioma tissues. GO analysis and KEGG pathway enrichment analysis were performed to explore potential biological functions of DEGs (Table S1). GO analysis showed that DEGs were significantly enriched in cell components such as centrosome, nucleocentric centromere and spindle tubulin and were involved in the biological processes such as cerebral cortex development, mitosis and DNA repair. KEGG pathway enrichment analysis showed that DEGs were mainly enriched in cell cycle, P53 signalling pathway, progesterone‐mediated oocyte maturation and ECM‐receptor interaction. Then, we selected twelve genes (*ASPM*, *CCNB2*, *CENPA*, *CENPF*, *COLA2*, *DLGAP5*, *GINS1*, *HSPG2*, *KLHDC8A*, *KIF14*, *LAMC1* and *RRM2*) that were not well studied at present, and verified the expression of these candidate genes by real‐time PCR in 30 clinical samples. It was found that *ASPM*, *CCNB2*, *HSPG2*, *KLHDC8A* and *RRM2* mRNA were differentially expressed in glioma tissues of different grades. And among them, ASPM changes most significantly (Figure S1). More importantly, further validation in the CGGA and TCGA‐LGG data sets and clinical samples also demonstrated that the expression of *ASPM* mRNA was increased with the increase in glioma grades, and the high expression of *ASPM* indicated a worse prognosis (Figure 1C,D). These results suggested that ASPM may act as a molecular marker for the diagnosis and prognosis prediction of glioma patients.

**TABLE 1:** Glioma differentially expressed genes screened using GSE4290, GSE4271 and GSE45921 data sets

	Differentially expressed genes (DEGs)
Up‐regulated genes	ADAM12, **ASPM,** AURKA, BIRC5, BRIP1, BUB1, BUB1B, C8orf4, CALU, CCNB2, CDC20, CDC45, CDK1, CDKN2C, CENPA, CENPF, CENPN, CENPU, CKS2, COL1A2, DLGAP5, DTL, FANCI, GINS1, GINS2, GTSE1, HMMR, HSPG2, JAG1, KIAA0101, KIF14, KIF20A, KIF23, KLHDC8A, LAMC1, MELK, MYBL2, NCAPG, NDC80, NUSAP1, PARPBP, PBK, RAD51AP1, RRM2, SERPINH1, SHCBP1, SHOX2, SOD2, TACC3, TIMP1, TOP2A
Down‐regulated genes	ADARB2, ADIRF, CNTN1, MAPK10, NTRK2, PPP1R1A, RTN1, SHANK2

### 3.2 ASPM inhibition affects proliferation, migration and invasion of glioma cells

**FIGURE 2:**
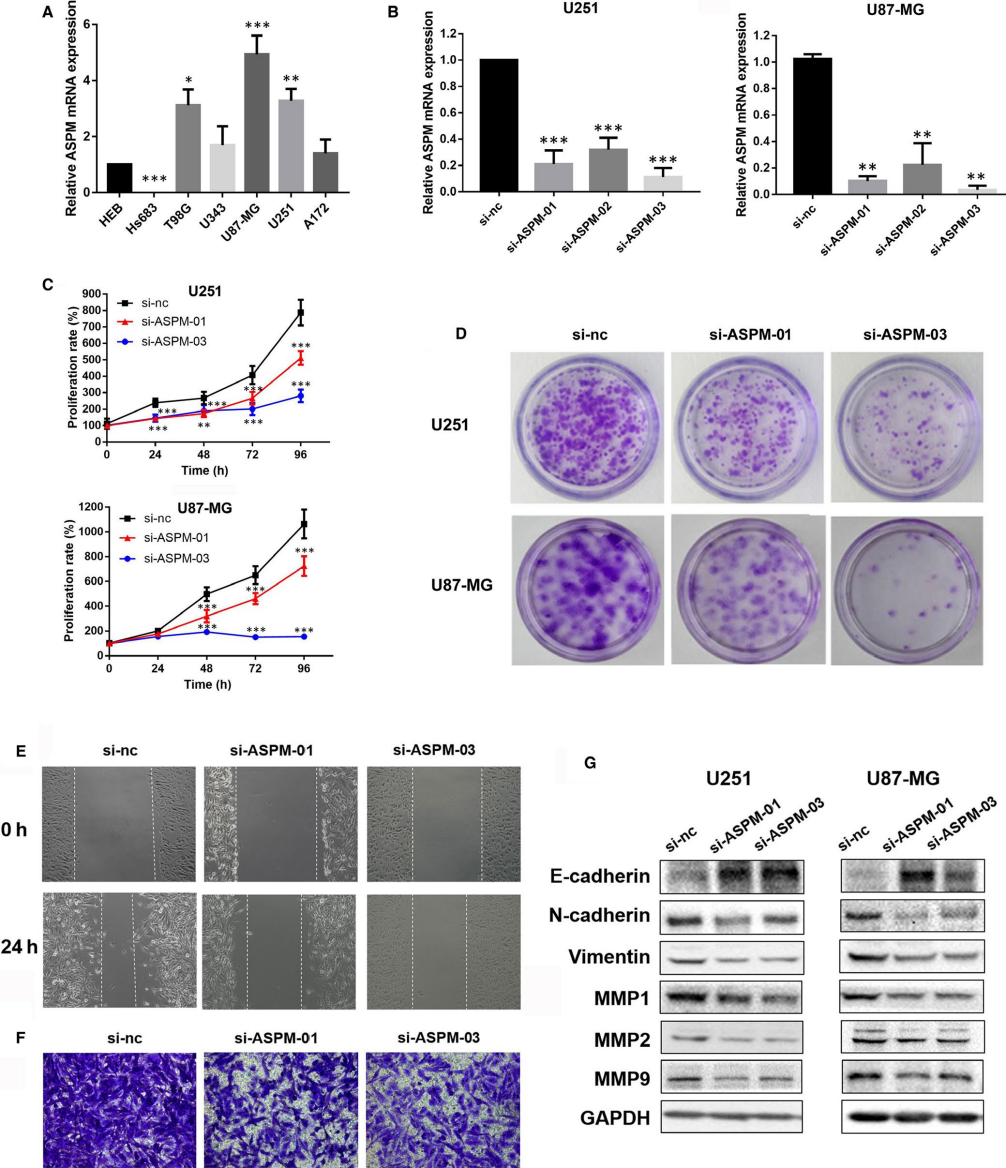
ASPM inhibition affects proliferation, migration and invasion of glioma cells. A, The expression of *ASPM* mRNA in different glioma cell lines; *P < 0.05 vs HEB, **P < 0.01 vs HEB, ***P < 0.001 vs HEB. The U87‐MG and U251 cells were transfected with a control siRNA or ASPM siRNA, (B) the mRNA expression of *ASPM* was evaluated by real‐time PCR, and (C, D) cell proliferation was measured by CCK8 assay and clonal formation assay; **P < 0.01 vs si‐nc group, ***P < 0.001 vs si‐nc group. E, Wound healing and (F) transwell migration tests were used to detect the migration ability of U251 and U87‐MG cell. G, The protein expression of E‐cadherin, N‐cadherin, Vimentin, MMP1, MMP2 and MMP9 in U87‐MG and U251 cells was measured by Western blot

In order to investigate the role of ASPM in glioma, we primarily detected the expression levels of *ASPM* mRNA in different glioma cell lines. The results showed that ASPM was highly expressed in U87‐MG and U251 cells and less expressed in Hs683 cells (Figure 2A). As shown in Figure 2C,D, CCK8 and colony formation results indicated that knockdown of ASPM by siRNA significantly inhibited the proliferation of U87‐MG and U251 cells compared with the si‐nc group. Highly aggressive and invasive nature were important malignant phenotypes of glioblastoma. Thus, we performed wound healing and transwell migration assays to evaluate the effect of ASPM on the migration ability of glioma cells. Obviously, inhibition of ASPM significantly reduced the migration of U87‐MG and U251 cells (Figure 2E,F). In addition, compared with the si‐nc group, E‐cadherin protein remarkably increased in the si‐ASPM treatment groups, whereas N‐cadherin, Vimentin, MMP1, MMP2 and MMP9 protein expression significantly decreased (Figure 2G). Collectively, these results indicated that down‐regulation of ASPM can inhibit the migration and invasion ability of glioma cells.

### 3.3 ASPM inhibition induces cell cycle arrest in the mitosis process of glioma cells

**FIGURE 3:**
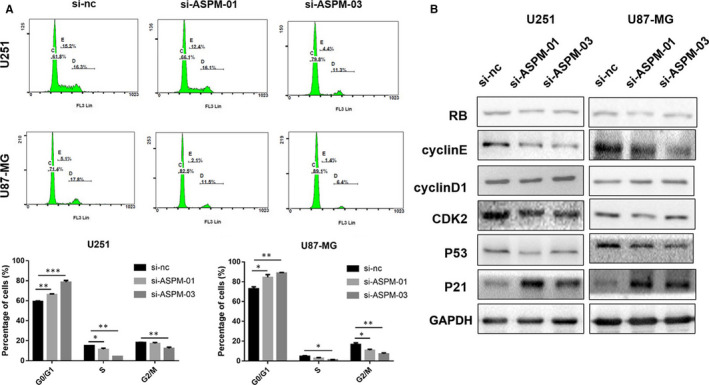
ASPM inhibition induces cell cycle arrest in the mitosis process of glioma cells. The U87‐MG and U251 cells were transfected with a control siRNA or ASPM siRNA for 72 h. A, B, Cell cycle was detected by flow cytometry, and the protein expression of RB, CyclinD1, CyclinE, CDK2, P53 and P21 was measured by Western blot

Studies have shown that ASPM participates in the regulation of symmetrical mitosis and the maintenance of neuronal granulosa cells by affecting the cell cycle.^6^, ^26^ However, whether ASPM is involved in the regulation of the cell cycle of glioma cells remains unclear. To investigate the role of ASPM in the cell cycle of glioma, flow cytometry was used to detect the cell cycle of U87‐MG and U251 cells transfected with si‐ASPM or si‐nc for 72 hours. As shown in Figure 3A, compared with the si‐nc group, ASPM ablation obviously decreased the proportion of cells in S phase and G2/M phase, whereas it increased the proportion of cells in G0/G1 phase. We then detected the effect of ASPM on the expression of cell cycle–related proteins, such as RB, CyclinE, CyclinD1, CDK2, P53 and P21, among which CyclinD1 is an early marker of G0/G1 phase and CyclinE is a late marker of G0/G1 phase. As shown in Figure 3B, inhibition of ASPM significantly decreased CyclinE protein expression, whereas it increased P21 protein expression, with the protein expression of RB, CyclinD1, CDK2 and P53 having no obvious change. Collectively, these data demonstrated that ASPM may lead to cell cycle arrest of glioma cells by causing abnormal expression of late‐G0/G1–related proteins.

### 3.4 FoxM1 expression is positively correlated with ASPM expression and predicts poor prognosis in gliomas

**FIGURE 4:**
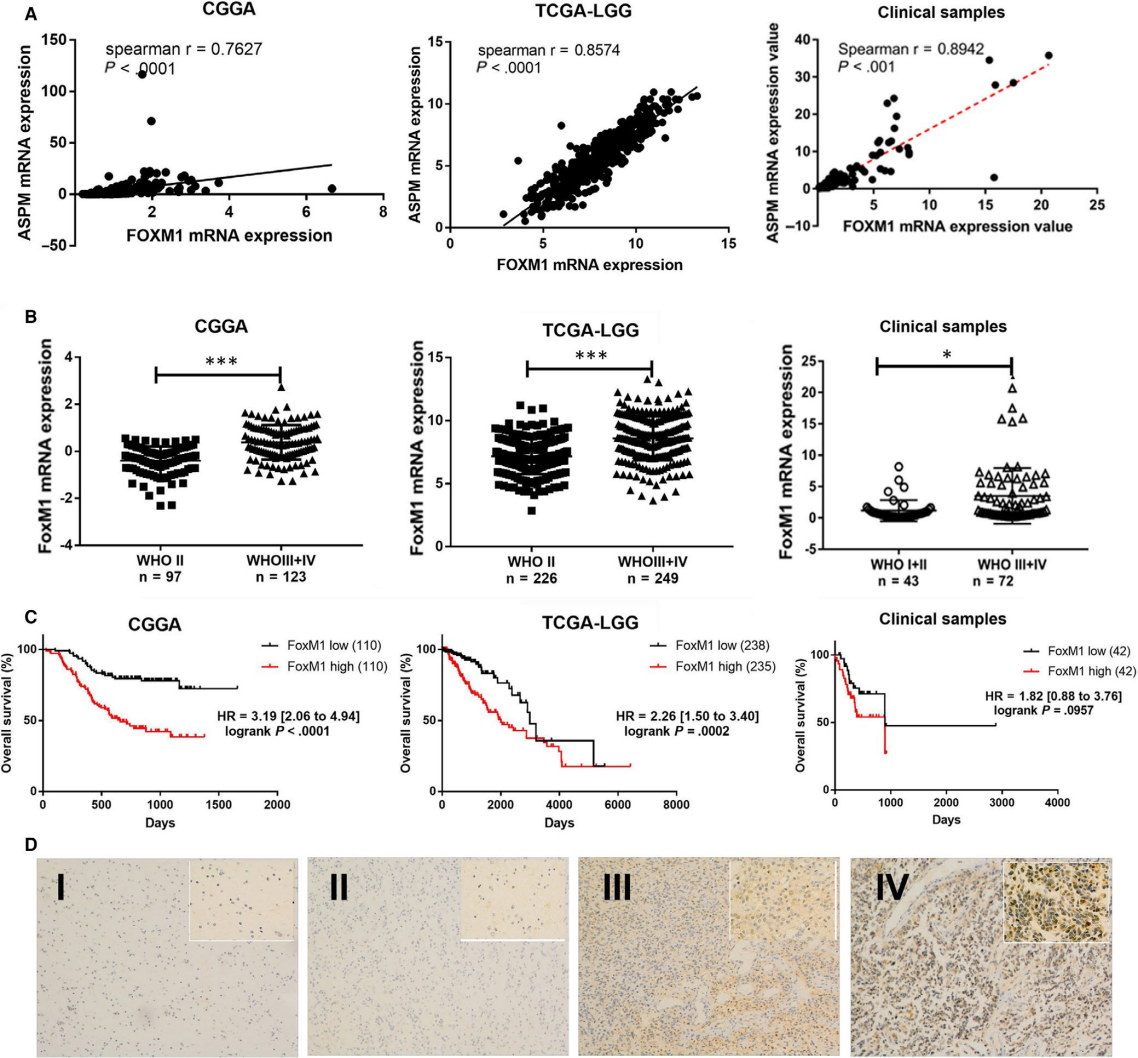
FoxM1 expression is positively correlated with ASPM expression and predicts poor prognosis in gliomas. A, The correlation between *FoxM1* and *ASPM* mRNA expression levels in CGGA data set, TCGA‐LGG data set and clinical samples. B, The expression of *FoxM1* mRNA in normal brain tissues and glioma tissues at different pathological grade in CGGA data set, TCGA‐LGG data set and clinical samples; *P < 0.05, ***P < 0.001. C, Kaplan‐Meier plot for overall survival between patients with high and low expression of *FoxM1* mRNA in CGGA data set, TCGA‐LGG data set and clinical samples. D, IHC detection of FoxM1 protein expression in glioma tissues. The sections were photographed at 100 × and 400 × magnification

To explore the mechanism of up‐regulation of ASPM expression in gliomas, we utilized the JASPAR database and ENCODE database to find the potential transcription factors that may regulate the expression of ASPM, and finally, we obtained 18 transcription factors (EGR1, ETS1, GATA3, NFYA, NFYB, POU2F2, YY1, RUNX3, SP1, SP2, STAT1, BRCA1, CHD1, E2F6, FoxM1, FOXO4, REST and STAT3). To further validate these findings, the correlation between the above transcription factors and ASPM was tested in the CGGA data set, TCGA data set and clinical samples, respectively. We found that *BRCA1*, *CHD1* and *FoxM1* mRNA expression was significantly positively correlated with *ASPM* mRNA expression in glioma tissues, and the highest correlation coefficient was found between *FoxM1* and *ASPM* expression (Figure S2, Figure 4A). In addition, the analysis of the CGGA data set, TCGA data set and clinical samples consistently suggested that the expression of FoxM1 was higher in gliomas and predicted a poor prognosis of glioma patients (Figure 4B,C).

### 3.5 Altering FoxM1 expression affects ASPM expression and proliferation, migration and invasion of glioma cells

**FIGURE 5:**
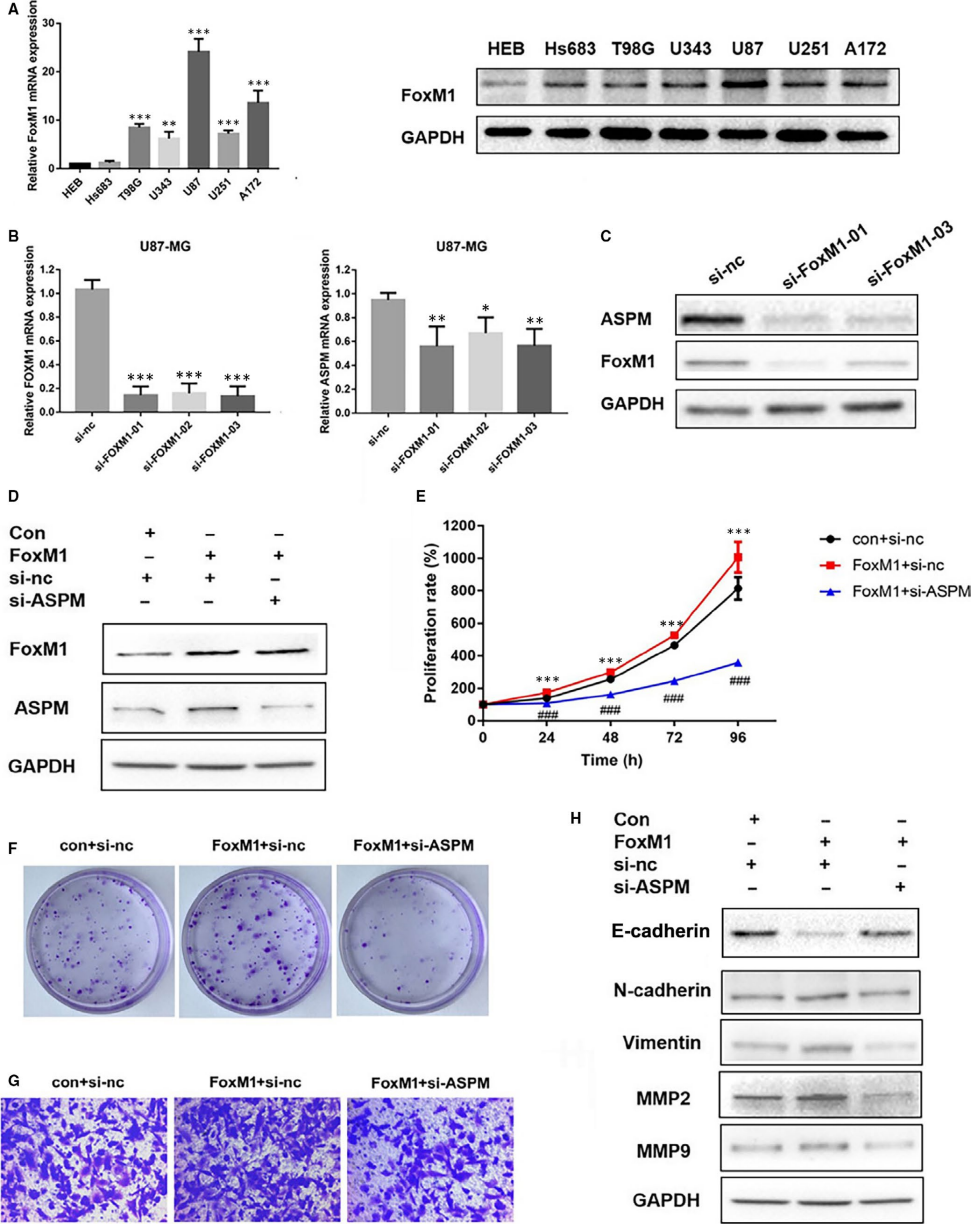
Altering FoxM1 expression affects ASPM expression, proliferation and migration of U251 cells. A, The expression of *FoxM1* mRNA and protein in different glioma cell lines; *P < 0.05 vs HEB, **P < 0.01 vs HEB, ***P < 0.001 vs HEB. B, C, The U87‐MG cells were transfected with a control siRNA or FoxM1 siRNA, and the mRNA and protein expression of FoxM1 and ASPM were measured by real‐time PCR and Western blot; **P < 0.01 vs si‐nc group, ***P < 0.001 vs si‐nc group. The U251 cells were transfected with FoxM1 overexpression vector and a control siRNA or ASPM siRNA. D, Protein expression of FoxM1 and ASPM was measured by Western blot. E, F, CCK8 assay and clonal formation assay was used to measure cell proliferation; ***P < 0.001 vs con group, ^###^ *P *< 0.001 vs FoxM1 + si‐nc group. G, Wound healing and transwell migration assays were used to detect the migration ability of U251 cells. H, The protein expression of E‐cadherin, N‐cadherin, Vimentin, MMP2 and MMP9 in U251 cells was measured by Western blot

Next, we further determined whether FoxM1 has a regulatory effect on ASPM expression. We examined the mRNA and protein expression levels of FoxM1 in different glioma cell lines and found that FoxM1 was highly expressed in U87‐MG cells and less expressed in Hs683 cells (Figure 5A). As shown in Figure 5B‐D, FoxM1 knockdown suppressed ASPM expression in U87‐MG, whereas FoxM1 overexpression increased ASPM expression in U251 cells. FoxM1 overexpression also promoted the proliferation and migration of U251 cells; however, ASPM knockdown reversed the effect induced by FoxM1 overexpression (Figure 5E‐G). In addition, FoxM1 overexpression also remarkably decreased E‐cadherin expression and increased N‐cadherin, Vimentin, MMP1, MMP2 and MMP9 expression, whereas FoxM1 overexpression–mediated changes of EMT‐related proteins were overturned by ASPM knockdown (Figure 5H). These results suggested that FoxM1 may regulate the expression of ASPM, thereby affecting the expression of EMT‐related proteins and ultimately regulating the migration and invasion of glioma cells.

### 3.6 FoxM1 transcriptionally regulates ASPM expression by directly binding to the promoter region

**FIGURE 6:**
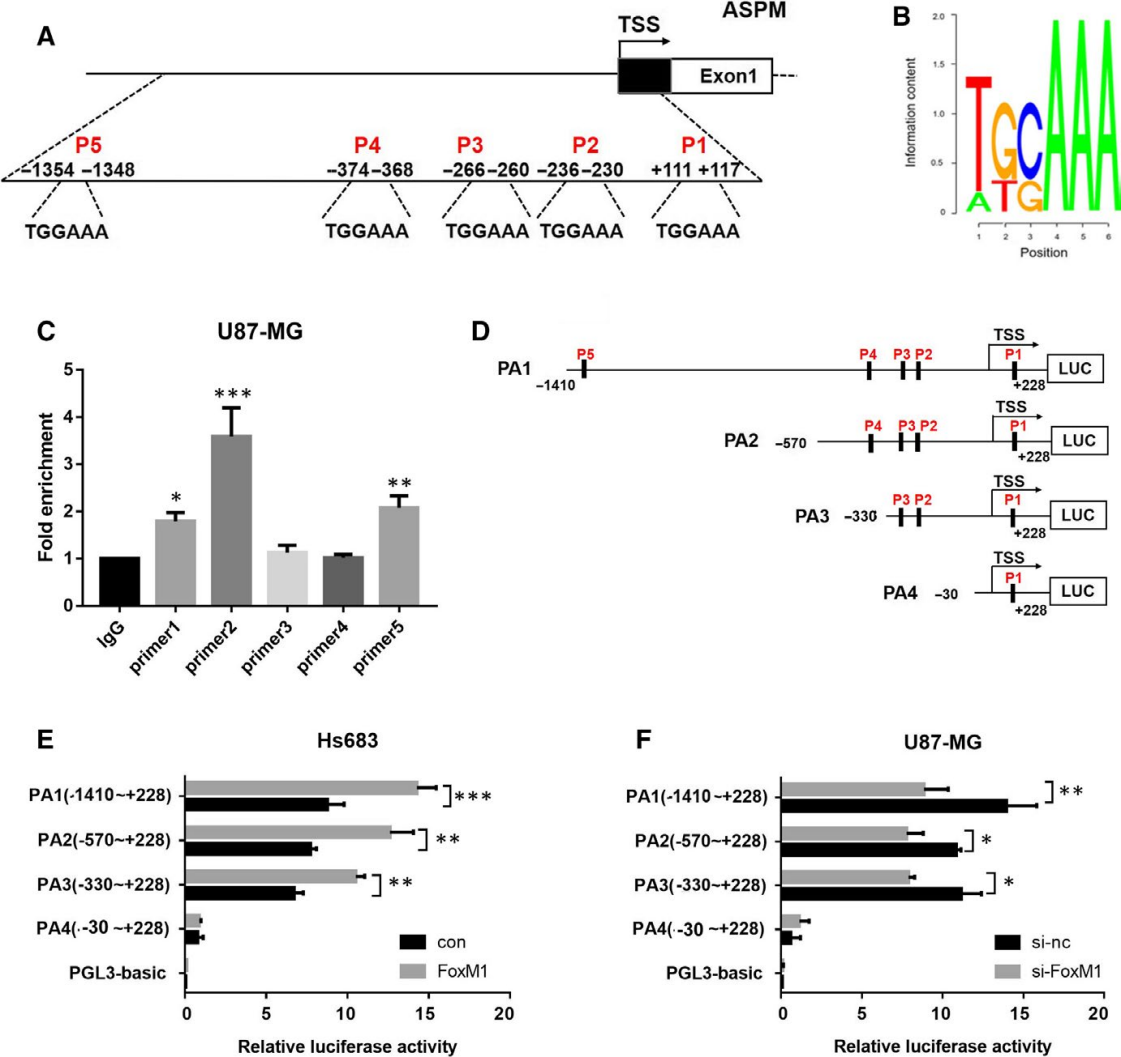
FoxM1 transcriptionally regulates ASPM expression by directly binding to the promoter region. A, Bioinformatics predicted the binding region between FoxM1 and ASPM promoter. B, The binding motif of FoxM1. C, ChIP assay was used to detect the binding of FoxM1 to ASPM promoter region in U87‐MG cells; *P < 0.05 vs IgG group, **P < 0.01 vs IgG group, ***P < 0.001 vs IgG group. D, The firefly luciferase reporter gene vectors with different lengths of ASPM promoter. E, Hs683 cells were transfected with ASPM promoter luciferase reporter gene vectors and FoxM1 overexpression vector for 24 h, and then, the dual‐luciferase activity was detected; **P < 0.01 vs con group, ***P < 0.001 vs con group. F, U87‐MG cells were transfected with ASPM promoter luciferase reporter gene vectors and FoxM1 siRNA for 24 h, and then, the dual‐luciferase activity was detected; *P < 0.05 vs si‐nc group, **P < 0.01 vs si‐nc group

To ascertain whether FoxM1 can directly bind to ASPM promoter region and thus regulate ASPM expression, we first used bioinformatics to predict the potential transcription factor binding sites of ASPM gene promoter and found that ASPM promoter region contains 5 potential FoxM1 binding sites (Figure 6A), and the binding motif of FoxM1 is TGCAAA (Figure 6B). Then, ChIP experiment was used to detect the direct binding of FoxM1 to ASPM promoter. As shown in Figure 6C, FoxM1 was strongly bound to ASPM promoter (−236 to ‐230 bp, −266 to ‐260 bp and −1354 to ‐1348 bp). To observe the effect of FoxM1 on the ASPM promoter activity, firefly luciferase reporter gene vectors containing different lengths of ASPM promoter fragments were constructed as shown in Figure 6D. In Hs683 cells, FoxM1 overexpression significantly activated the luciferase activity of full‐length ASPM promoter PA1, whereas its regulatory effect on the truncated ASPM promoter PA4 completely disappeared (Figure 6E). In U87‐MG cells, FoxM1 knockdown remarkably suppressed the luciferase activity of full‐length ASPM promoter PA1 and truncated ASPM promoters PA2 and PA3, whereas it had no regulatory effect on truncated ASPM promoter PA4 (Figure 6F). These results demonstrated that FoxM1 mainly regulates ASPM promoter activity through binding to ASPM promoter regions (−236 to ‐230 bp) and (−1354 to ‐1348 bp).

### 3.7 ASPM is essential for the growth of subcutaneous tumours in nude mice

**FIGURE 7:**
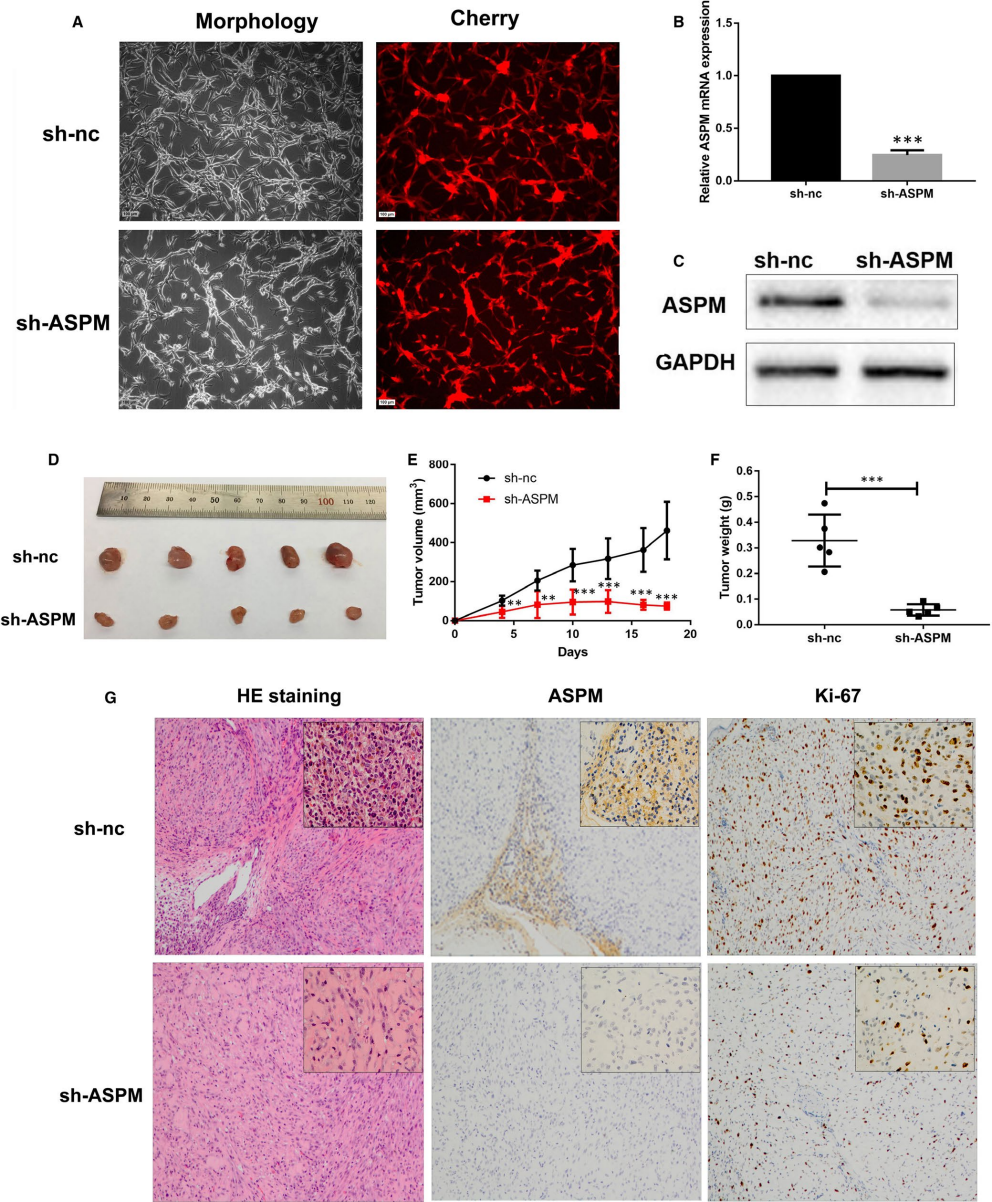
ASPM is essential for the growth of subcutaneous tumours in nude mice. A, The ASPM stable knockout U87‐MG cells (U87‐MG‐sh‐ASPM cells) and the control U87‐MG cells (U87‐MG‐sh‐nc cells) were imaged using fluorescence microscopy. B, C, The mRNA and protein expression of ASPM were measured by real‐time PCR and Western blot; ***P < 0.001 vs U87‐MG‐sh‐nc cells. D, Subcutaneous tumour was stripped and photographed after transplantation for 18 days. E, Subcutaneous tumour growth curve of nude mice. F, Subcutaneous tumour weight in nude mice. G, The histopathological characteristics and the expression levels of ASPM and Ki‐67 protein in subcutaneous tumours of nude mice were detected by HE staining and IHC, respectively

A xenograft nude mouse model was established to evaluate the effect of ASPM on the growth of gliomas in vivo. Firstly, we used lentiviral infection to establish ASPM stable knockdown U87‐MG cells. As shown in Figure 7A, the fluorescence‐positive rate of cells was >90% after lentiviral infection. In agreement, compared with U87‐MG‐sh‐nc cells, ASPM expression was significantly lower in U87‐sh‐ASPM cells (Figure 7B,C). In the subcutaneous tumorigenesis model of nude mice, the tumours formed by U87‐sh‐ASPM cells were significantly smaller than those formed by U87‐sh‐nc cells, and the proliferation rate of tumours in vivo was also remarkably lower (Figure 7D‐F). IHC results showed that Ki‐67‐positive cells in the tumour tissues formed by U87‐sh‐ASPM cells were significantly less than those formed by U87‐sh‐ASPM (Figure 7G). This evidence indicated that inhibition of ASPM can repress the formation and growth of subcutaneous tumours.

## 4 DISCUSSION

In this study, GSE4290, GSE4271 and GSE45921 were used to screen out 59 differentially expressed genes in normal brain tissues and gliomas of different grades. Through verification of the CGGA data set, TCGA‐LGG data set and clinical samples, we found that the expression of ASPM mRNA increased with the increase of glioma grade, and the high expression of ASPM predicted worse prognosis, suggesting that ASPM may function as a molecular marker for the diagnosis and prognosis prediction of glioma patients.

ASPM plays an important role in controlling the neurogenesis and cerebral cortical size, and its homozygous mutation can lead to apoptosis of neural progenitor cells and microcephaly.^5^, ^27^ Previous studies have reported that ASPM is mainly distributed in the centrosomes, spindle microtubule intermediates and mid‐body^28^, ^29^ and plays a vital role in cell division and proliferation in foetal tissues and human cancer cells.^30^, ^31^ In the development of mouse brain, ASPM maintains the symmetrical division and proliferation of embryonic neuroepithelial cells by controlling mitotic spindle orientation.^6^ In gliomas, ASPM is elevated in glioma tissues, and ASPM knockdown significantly represses the proliferation of glioma spheres.^12^, ^13^ Consistent with previous studies, our results also indicated that ASPM is significantly positively correlated with the pathological grade of glioma and poor prognosis of patients, and knockdown of ASPM could inhibit the proliferation of glioma cells.

Previous studies have found that high expression of ASPM is significantly positively correlated with vascular invasion and early metastasis of hepatocellular carcinoma, and its high expression indicates poor prognosis of hepatocellular carcinoma patients.^32^ In metastatic melanoma tissues, ASPM gene is highly expressed, and ASPM overexpression improves the invasion ability of melanoma cells,^33^ indicating that ASPM may mediate the invasion and metastasis of tumours. However, the effect of ASPM on the migration and invasion ability of glioma cells has not been reported. In the present study, we found that knockdown of ASPM significantly reduced the migration ability of glioma cells. EMT plays an important role in the invasion and migration of gliomas,^34^ and MMPs can promote the migration, invasion and distant metastasis of tumour cells to surrounding tissues by degrading the extracellular matrix.^35^ In this study, we found for the first time that knockdown of ASPM can increase the expression levels of E‐cadherin protein in glioma cells and decrease the expression levels of N‐cadherin and Vimentin, MMP1, MMP2 and MMP9 proteins, suggesting that ASPM may affect the migration and invasion of glioma cells by regulating the expression of EMT‐related proteins.

Abnormal proliferation of tumour cells is often accompanied by abnormal expression of cell cycle regulators.^36^ Previous studies have shown that ASPM is involved in cell cycle regulation.^6^, ^26^, ^29^ For example, HCV NS5A protein down‐regulates the protein expression of ASPM and induces aberrant mitotic cell cycle through the PKR‐p38 signalling pathway.^37^ In mouse neuron stem cells, full length of ASPM inhibits CyclinE ubiquitination in the form of a complex with CDK2/CyclinE, thereby affecting cell cycle duration and participating in stem cell maintenance.^26^ In the current work, we found that knockdown of ASPM can induce cell arrest at G0/G1 phase and down‐regulate CyclinE expression in glioma cells. CyclinE/CDK2 complex regulates the phosphorylation of RB, thereby functioning as the G1 checkpoint.^38^ In addition, it has been reported that ASPM promotes the ubiquitination modification and inhibits the degradation of CyclinE,^26^ suggesting that ASPM interference may lead to the cell cycle arrest of glioma cells by regulating the abnormal protein expression at the late G0/G1 phase.

At present, the mechanism that regulates the abnormal expression of ASPM in gliomas remains unrevealed. Studies have found that FoxOs can directly inhibit ASPM expression and thus regulate the proliferation of neural stem cells.^39^ EGFRvIII can up‐regulate the expression of ASPM, whereas the overexpression of EGFR inhibitor erlotinib and anti‐oncogene PTEN can reverse this effect, indicating that ASPM expression may be regulated by the PI3K pathway.^13^ In order to further investigate the mechanism that induced ASPM up‐regulation in gliomas, we utilized the JASPAR and ENCODE database prediction combined with clinical samples and the CGGA and TCGA database validation, and first discovered FoxM1 may be a potential control ASPM expression transcription factors.

FoxM1, a proliferation‐specific transcription factor, was first discovered in the cervical cancer cell line HeLa.^14^ FoxM1 is highly expressed in embryonic mouse tissues with a high proliferation index, such as thymus, small intestine and testicles, and it is also elevated in a variety of tumour tissues and is associated with tumour malignancy and patient prognosis.^17^, ^18^, ^40^, ^41^ It has been reported that FoxM1 is overexpressed in GBM and suggests poor prognosis in glioma patients.^19^, ^42^ Similar to previous studies, we also found that FoxM1 is highly expressed in gliomas and its high expression predicts the poor prognosis of glioma patients. In addition, studies have demonstrated that FoxM1 is involved in maintaining tumorigenicity of GBM stem cells, and its mechanism may be related to FoxM1 promoting nuclear localization of beta‐catenin.^43^ However, whether FoxM1 plays a role in tumour development by transcriptional regulation of ASPM expression is still unknown. In this study, we demonstrated for the first time that FoxM1 can directly regulate ASPM transcription in glioma cells, thereby affecting their expression and thereby regulating the proliferation, migration and invasion of glioma cells.

In summary, the present study is the first to reveal that ASPM promoted glioma cell proliferation, migration and invasion and tumour growth, which is mediated by the transcriptional activation of FoxM1. In the present study, using the TCGA and CGGA data set analysis, we found that ASPM was highly correlated with FoxM1 expression. Due to the presence of tumour heterogeneity, FoxM1 was unfortunately present only in the DEG list of GSE4290 and GSE45921, and therefore not in the list of 59 DEGs we finally found. This phenomenon suggested that using multiple data sets to screen for DEGs may yield more reliable results but may also lose valuable information. It has been confirmed that FoxM1 is involved in chemotherapy resistance in a variety of tumours.^20^, ^44^, ^45^, ^46^ Moreover, studies have shown that FoxM1 can directly promote the expression of RFC5 at the transcription level, thus leading to TMZ resistance independent of MGMT activity.^20^ Knockdown of FoxM1 can inhibit the expression of Rad51 and increase the TMZ sensitivity of recurrent GBM cells.^46^ These studies suggest that FoxM1 is involved in the regulation of TMZ resistance and drug resistance in glioma. In the future, more experiments are needed to explore the role of the FoxM1‐ASPM axis in TMZ chemotherapy resistance.

## CONFLICT OF INTEREST

The authors declare no conflict of interest.

## AUTHOR CONTRIBUTIONS

W.J. Zeng and Q. Cheng performed the experiments and wrote the paper. Z.P. Wen and J.Y. Wang performed data analysis. Y.H. Chen and J. Zhao collected the clinical samples and performed follow‐up. Z.C. Gong and X.P. Chen designed the study. All authors reviewed and approved the final version of the manuscript.

## Supporting information

Figure S1

Figure S1

Table S1

Table S2

Table S3

## Data Availability

The data that support the findings described in the current study are available in the article.

## References

[1] Louis DN , Ohgaki H , Wiestler OD , et al. The 2007 WHO classification of tumours of the central nervous system. Acta Neuropathol. 2007;114:97‐109. 17618441 10.1007/s00401-007-0243-4 PMC1929165

[2] Louis DN , Perry A , Reifenberger G , et al. The 2016 World Health Organization Classification of Tumors of the Central Nervous System: a summary. Acta Neuropathol. 2016;131:803‐820. 27157931 10.1007/s00401-016-1545-1

[3] Nabors LB , Portnow J , Ammirati M , et al. Central nervous system cancers, version 2.2014. Featured updates to the NCCN Guidelines. J Natl Compr Canc Netw. 2014;12:1517‐1523. 25361798 10.6004/jnccn.2014.0151 PMC4337873

[4] Nabors LB , Portnow J , Ammirati M , et al. Nervous system cancers, version 1.2015. J Natl Compr Canc Netw. 2015;13:1191‐1202. 26483059 10.6004/jnccn.2015.0148

[5] Shen J , Eyaid W , Mochida GH , et al. ASPM mutations identified in patients with primary microcephaly and seizures. J Med Genet. 2005;42:725‐729. 16141009 10.1136/jmg.2004.027706 PMC1736131

[6] Fish JL , Kosodo Y , Enard W , et al. Aspm specifically maintains symmetric proliferative divisions of neuroepithelial cells. Proc Natl Acad Sci U S A. 2006;103:10438‐10443. 16798874 10.1073/pnas.0604066103 PMC1502476

[7] Wakefield JG , Bonaccorsi S , Gatti M . The drosophila protein asp is involved in microtubule organization during spindle formation and cytokinesis. J Cell Biol. 2001;153:637‐648. 11352927 10.1083/jcb.153.4.637 PMC2192390

[8] Novorol C , Burkhardt J , Wood KJ , et al. Microcephaly models in the developing zebrafish retinal neuroepithelium point to an underlying defect in metaphase progression. Open Biol. 2013;3:130065. 24153002 10.1098/rsob.130065 PMC3814721

[9] Vulcani‐Freitas TM , Saba‐Silva N , Cappellano A , et al. ASPM gene expression in medulloblastoma. Childs Nerv Syst. 2011;27:71‐74. 20694558 10.1007/s00381-010-1252-5

[10] Wang W , Hsu C , Wang T , et al. A gene expression signature of epithelial tubulogenesis and a role for ASPM in pancreatic tumor progression. Gastroenterology. 2013;145:1110‐1120. 23896173 10.1053/j.gastro.2013.07.040

[11] Pai VC , Hsu CC , Chan TS , et al. ASPM promotes prostate cancer stemness and progression by augmenting Wnt‐Dvl‐3‐beta‐catenin signaling. Oncogene. 2018;8:1354. 10.1038/s41388-018-0561-0 30390070

[12] Bikeye S‐N , Colin C , Marie Y , et al. ASPM‐associated stem cell proliferation is involved in malignant progression of gliomas and constitutes an attractive therapeutic target. Cancer Cell Int. 2010;10:1. 20142996 10.1186/1475-2867-10-1 PMC2817685

[13] Horvath S , Zhang B , Carlson M , et al. Analysis of oncogenic signaling networks in glioblastoma identifies ASPM as a molecular target. Proc Natl Acad Sci U S A. 2006;103:17402‐17407. 17090670 10.1073/pnas.0608396103 PMC1635024

[14] Wierstra I . The transcription factor FOXM1 (Forkhead box M1): proliferation‐specific expression, transcription factor function, target genes, mouse models, and normal biological roles. Adv Cancer Res. 2013;118:97‐398. 23768511 10.1016/B978-0-12-407173-5.00004-2

[15] Laoukili J , Kooistra MRH , Brás A , et al. FoxM1 is required for execution of the mitotic programme and chromosome stability. Nat Cell Biol. 2005;7:126‐136. 15654331 10.1038/ncb1217

[16] Yuan B , Liu Y , Yu X , et al. FOXM1 contributes to taxane resistance by regulating UHRF1‐controlled cancer cell steness. Cell Death & Disease. 2018;9(5):562–573. 10.1038/s41419-018-0631-9. 29752436 PMC5948215

[17] Wierstra I . FOXM1 (Forkhead box M1) in tumorigenesis: overexpression in human cancer, implication in tumorigenesis, oncogenic functions, tumor‐suppressive properties, and target of anticancer therapy. Adv Cancer Res. 2013;119:191‐419. 23870513 10.1016/B978-0-12-407190-2.00016-2

[18] Nandi D , Cheema PS , Jaiswal N , et al. FoxM1: repurposing an oncogene as a biomarker. Semin Cancer Biol. 2018;52:74‐84. 28855104 10.1016/j.semcancer.2017.08.009

[19] Dai B , Kang S‐H , Gong W , et al. Aberrant FoxM1B expression increases matrix metalloproteinase‐2 transcription and enhances the invasion of glioma cells. Oncogene. 2007;26:6212‐6219. 17404569 10.1038/sj.onc.1210443

[20] Peng W‐X , Han X , Zhang C‐L , et al. FoxM1‐mediated RFC5 expression promotes temozolomide resistance. Cell Biol Toxicol. 2017;33:527‐537. 28185110 10.1007/s10565-017-9381-1

[21] Gao YF , Zhu T , Mao CX , et al. PPIC, EMP3 and CHI3L1 Are Novel Prognostic Markers for High Grade Glioma. Int J Mol Sci. 2016;17(11):1808–1821. 10.3390/ijms17111808 PMC5133809 27801851

[22] Zhang X , Lv Q‐L , Huang Y‐T , et al. Akt/FoxM1 signaling pathway‐mediated upregulation of MYBL2 promotes progression of human glioma. J Exp Clin Cancer Res. 2017;36:105. 28784180 10.1186/s13046-017-0573-6 PMC5547476

[23] Lv Q‐L , Hu L , Chen S‐H , et al. A long noncoding RNA ZEB1‐AS1 promotes tumorigenesis and predicts poor prognosis in glioma. Int J Mol Sci. 2016;17:1431. 10.3390/ijms17091431 PMC5037710 27589728

[24] Wen ZP , Zeng WJ , Chen YH , et al. Knockdown ATG4C inhibits gliomas progression and promotes temozolomide chemosensitivity by suppressing autophagic flux. Journal of Experimental & Clinical Cancer Research. 2019;38(1):298–313. 10.1186/s13046-019-1287-8. 31291988 PMC6617611

[25] Naito S , von Eschenbach AC , Giavazzi R , et al. Growth and metastasis of tumor cells isolated from a human renal cell carcinoma implanted into different organs of nude mice. Cancer Res. 1986;46:4109‐4115. 3731078

[26] Capecchi MR , Pozner A . ASPM regulates symmetric stem cell division by tuning Cyclin E ubiquitination. Nat Commun. 2015;6:8763. 26581405 10.1038/ncomms9763 PMC5025044

[27] Bond J , Roberts E , Mochida GH , et al. ASPM is a major determinant of cerebral cortical size. Nat Genet. 2002;32:316‐320. 12355089 10.1038/ng995

[28] Higgins J , Midgley C , Bergh A‐M , et al. Human ASPM participates in spindle organisation, spindle orientation and cytokinesis. BMC Cell Biol. 2010;11:85. 21044324 10.1186/1471-2121-11-85 PMC2988714

[29] Paramasivam M , Chang YJ , LoTurco JJ . ASPM and citron kinase co‐localize to the midbody ring during cytokinesis. Cell Cycle. 2007;6:1605‐1612. 17534152 10.4161/cc.6.13.4356

[30] Kouprina N , Pavlicek A , Collins NK , et al. The microcephaly ASPM gene is expressed in proliferating tissues and encodes for a mitotic spindle protein. Hum Mol Genet. 2005;14:2155‐2165. 15972725 10.1093/hmg/ddi220

[31] Williams SE , Garcia I , Crowther AJ , et al. Aspm sustains postnatal cerebellar neurogenesis and medulloblastoma growth in mice. Development. 2015;142:3921‐3932. 26450969 10.1242/dev.124271 PMC4712878

[32] Lin S‐Y , Pan H‐W , Liu S‐H , et al. ASPM is a novel marker for vascular invasion, early recurrence, and poor prognosis of hepatocellular carcinoma. Clin Cancer Res. 2008;14:4814‐4820. 18676753 10.1158/1078-0432.CCR-07-5262

[33] Kabbarah O , Nogueira C , Feng B , et al. Integrative genome comparison of primary and metastatic melanomas. PLoS One. 2010;5:e10770. 20520718 10.1371/journal.pone.0010770 PMC2875381

[34] Meel MH , Schaper SA , Kaspers GJL , et al. Signaling pathways and mesenchymal transition in pediatric high‐grade glioma. Cell Mol Life Sci. 2018;75:871‐887. 29164272 10.1007/s00018-017-2714-7 PMC5809527

[35] Glentis A , Oertle P , Mariani P , et al. Cancer‐associated fibroblasts induce metalloprotease‐independent cancer cell invasion of the basement membrane. Nat Commun. 2017;8:924. 29030636 10.1038/s41467-017-00985-8 PMC5640679

[36] Kastan MB , Bartek J . Cell‐cycle checkpoints and cancer. Nature. 2004;432:316‐323. 15549093 10.1038/nature03097

[37] Wu SC , Chang SC , Wu HY , et al. Hepatitis C virus NS5A protein down‐regulates the expression of spindle gene Aspm through PKR‐p38 signaling pathway. J Biol Chem. 2008;283:29396‐29404. 18728014 10.1074/jbc.M802821200 PMC2662026

[38] Koff A , Giordano A , Desai D , et al. Formation and activation of a cyclin E‐cdk2 complex during the G1 phase of the human cell cycle. Science. 1992;257:1689‐1694. 1388288 10.1126/science.1388288

[39] Paik J‐H , Ding Z , Narurkar R , et al. FoxOs cooperatively regulate diverse pathways governing neural stem cell homeostasis. Cell Stem Cell. 2009;5:540‐553. 19896444 10.1016/j.stem.2009.09.013 PMC3285492

[40] Hu C , Liu D , Zhang Y , et al. LXRalpha‐mediated downregulation of FOXM1 suppresses the proliferation of hepatocellular carcinoma cells. Oncogene. 2014;33:2888‐2897. 23812424 10.1038/onc.2013.250

[41] Teh MT , Wong ST , Neill GW , et al. FOXM1 is a downstream target of Gli1 in basal cell carcinomas. Cancer Res. 2002;62:4773‐4780. 12183437

[42] Gong A , Huang S . FoxM1 and Wnt/beta‐catenin signaling in glioma stem cells. Cancer Res. 2012;72:5658‐5662. 23139209 10.1158/0008-5472.CAN-12-0953 PMC3500394

[43] Zhang N , Wei P , Gong A , et al. FoxM1 promotes beta‐catenin nuclear localization and controls Wnt target‐gene expression and glioma tumorigenesis. Cancer Cell. 2011;20:427‐442. 22014570 10.1016/j.ccr.2011.08.016 PMC3199318

[44] Hu C‐J , Wang B , Tang BO , et al. The FOXM1‐induced resistance to oxaliplatin is partially mediated by its novel target gene Mcl‐1 in gastric cancer cells. Biochim Biophys Acta. 2015;1849:290‐299. 25482013 10.1016/j.bbagrm.2014.11.008

[45] Lin J , Zheng Y , Chen K , et al. Inhibition of FOXM1 by thiostrepton sensitizes medulloblastoma to the effects of chemotherapy. Oncol Rep. 2013;30:1739‐1744. 23912794 10.3892/or.2013.2654

[46] Zhang N , Wu X , Yang L , et al. FoxM1 inhibition sensitizes resistant glioblastoma cells to temozolomide by downregulating the expression of DNA‐repair gene Rad51. Clin Cancer Res. 2012;18:5961‐5971. 22977194 10.1158/1078-0432.CCR-12-0039 PMC3639123

