# Supplementary figures and images for: Aberrant ASPM expression mediated by transcriptional regulation of FoxM1 promotes the progression of gliomas
Source: J Cell Mol Med. 2020 Jul 15;24(17):9613–26. doi: 10.1111/jcmm.15435 (PMC7520292; doi:10.1111/jcmm.15435)

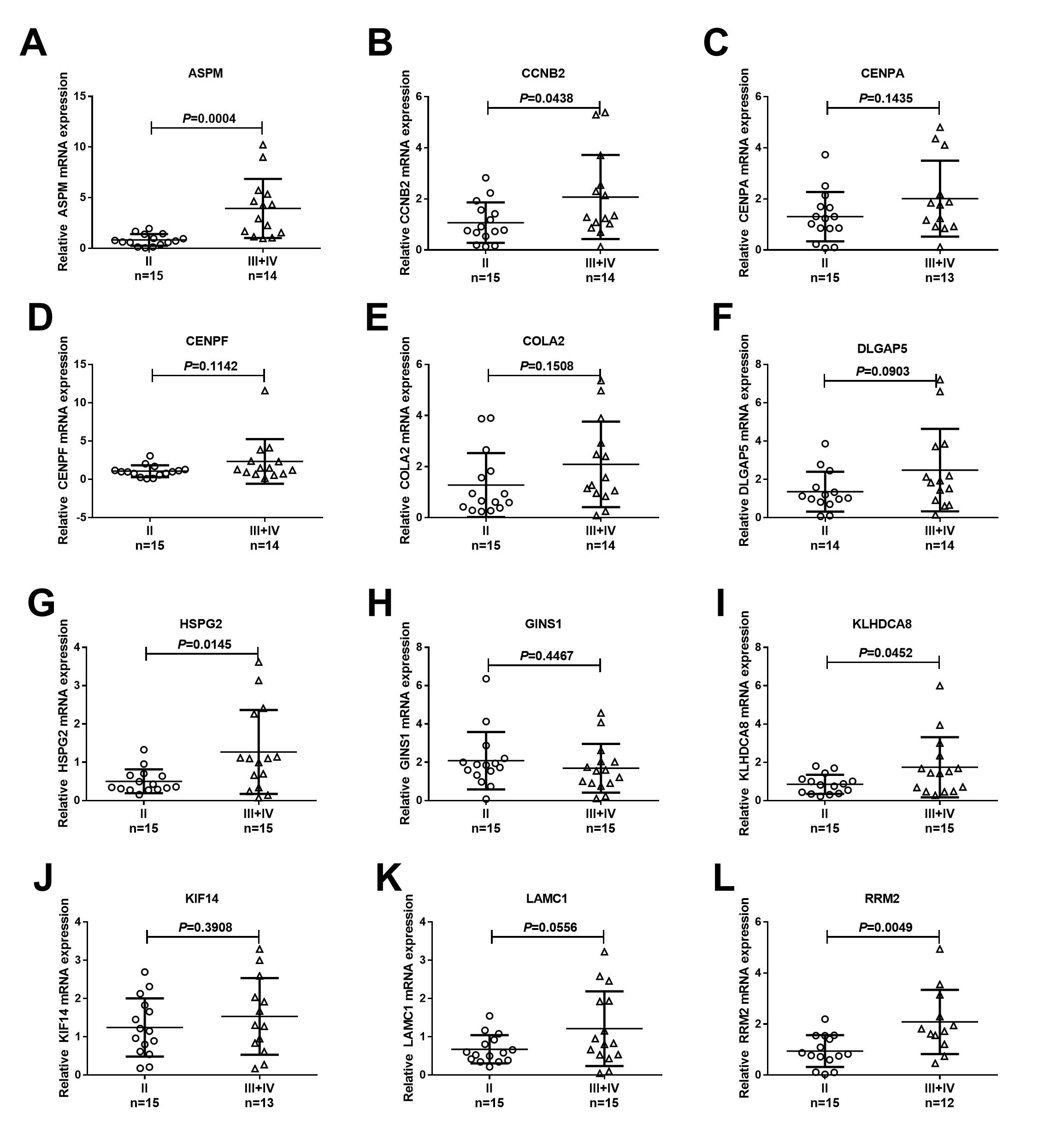

Supplement: Supplementary file 1 — Figure S1 [file JCMM-24-9613-s001.jpg]

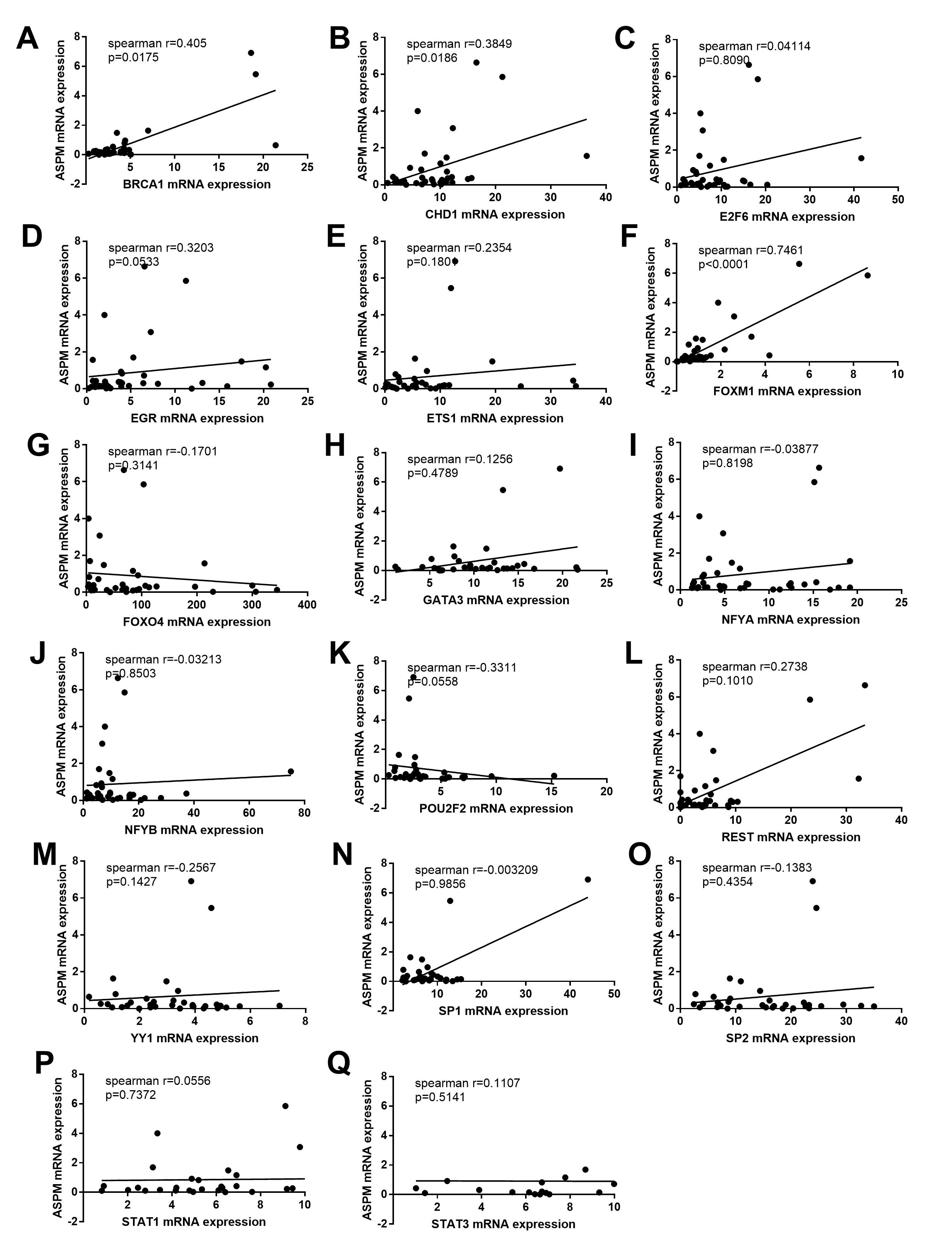

Supplement: Supplementary file 2 — Figure S1 [file JCMM-24-9613-s002.jpg]
