# Supplementary material for: Aberrant ASPM expression mediated by transcriptional regulation of FoxM1 promotes the progression of gliomas
Source: J Cell Mol Med. 2020 Jul 15;24(17):9613–26. doi: 10.1111/jcmm.15435 (PMC7520292; doi:10.1111/jcmm.15435)
Supplement: Supplementary file 3 — Table S1 [file JCMM-24-9613-s003.docx]

| **Supplementary Table 1. Real-time PCR primers for the detection of gene expression.** | | |
| --- | --- | --- |
| **Gene symbol** | **Forward primer (5’-3’)** | **Reverse primer (5’-3’)** |
| ASPM | TCCCGTCACCTTGGCTTATT | TGCCGGAATCCTGAGTTTCT |
| CCNB2 | CTGGAGGTTTTGCAGTCCAT | GAAGCCAAGAGCAGAGCAGT |
| CENPA | TCCTCCCATCAACACAGTCG | GGCTTGCCAATTGAAGTCCA |
| CENPF | ACCGAGGGTACAAACCTGAA | GCAGCTTGTTGGCTTCTTTC |
| COLA2 | GCAACCTGAAAAAGGCTGTC | GGCGTGATGGCTTATTTGTT |
| DLGAP5 | CCAGTCGACACAGGAAGGAT | CATTGCCCTTGGCTTAACAT |
| GINS1 | GAAATCGACGCTGCACTGTA | TTCATCTCCTCCCAGTGACC |
| HSPG2 | ATGCTGGGGTCTACATTTGC | ATAGGCTGGGGACTTGCTTT |
| KLHDC8A | GCCTTCTCCAGCTTTGTGAC | CTCCAGGACAGTGGGTTGAT |
| KIF14 | TCGGGTTCGTAACCTGAAAC | TGTAATGTCGGGTTCCCATT |
| LAMC1 | TCCAGCAGACAAATGCAAAG | TCACACCTCTCACAGCCTTG |
| RRM2 | ACAGAAGCCCGCTGTTTCTA | CCCAGTCTGCCTTCTTCTTG |
| GAPDH | CTGCACCACCAACTGCTTAG | AGGTCCACCACTGACACGTT |
| BRCA1 | TGAAGAAAGAGGAACGGGCT | TGGCTCCCATGCTGTTCTAA |
| CHD1 | TGAAGGGTCCAACATTCCGA | AATGAGCTGCCTTTGTGTGG |
| E2F6 | GGCCTTCCATGAACAGATCG | CAGATGAAGAGGTCCCGACA |
| EGR1 | AGCAGCACCTTCAACCCTC | AAAGCGGCCAGTATAGGTGA |
| ETS1 | GACCCTCTCCAGACAGACAC | GTCCTCTGAGTCGAAGCTGT |
| FOXM1 | AGTTCTGATGGACTGGGCTC | CTCTCAGTGCTGTTGATGGC |
| FOXO4 | GGAGAACCTGGAGTGTGACA | AGACCCCACCCTAGCTCTAA |
| GATA3 | AGGGAGTGTGTGAACTGTGG | ATTGGCATTCCTCCTCCAGA |
| NFYA | GGAGGGATGGTCATGATGGT | TGCCTCTAGTTTAGCTCGGG |
| NFYB | GGTGCCATCAAGAGAAACGG | GTGACTGCTCCACCAATTCC |
| POU2F2 | AGGACCAGCATCGAGACAAA | CAGGGGTTGATGCGTTTCTC |
| YY1 | GTTCAGGGATAACTCGGCCA | TTCGAACGTGCACTGAAAGG |
| RUNX3 | CCTTCAAGGTGGTGGCATTG | AACACAGTGATGGTCAGGGT |
| REST | TGCGCGAATACAGTTATGGC | ACCAGGTAATCACAGCAGCT |
| SP1 | CCCACAAGCCCAAACAATCA | TGCACCTGGATTCCTGAAGT |
| SP2 | CTGCTCATTGTTCAGAGCCC | AGCTGCCTGGATCTGAAAGT |
| STAT1 | ATGCTTGCTTGGATCAGCTG | GGAAAAGACTGAAGGTGCGG |
| STAT3 | AAAGCAGCAAAGAAGGAGGC | CTGGCCGACAATACTTTCCG |
|  |  |  |
