# Supplementary material for: Aberrant ASPM expression mediated by transcriptional regulation of FoxM1 promotes the progression of gliomas
Source: J Cell Mol Med. 2020 Jul 15;24(17):9613–26. doi: 10.1111/jcmm.15435 (PMC7520292; doi:10.1111/jcmm.15435)
Supplement: Supplementary file 4 — Table S2 [file JCMM-24-9613-s004.docx]

| **Supplementary Table 2. Real-time PCR primers for the detection of ASPM promoter.** | | |
| --- | --- | --- |
| Primer name | Forward primer (5’-3’) | Reverse primer (5’-3’) |
| Primer1 (+111~+117) | TGCGAGTTTATTGGGCTTGT | GATCCGGGACTTACGCTGA |
| Primer2 | TGGAAACCGCAATGATTACTACA | ACCCGGGGCTTAGAATTGAA |
| (-236~-230) |  |  |
| Primer3 | GGCGGGGTATGCATATGAGA | ACCCGGGGCTTAGAATTGAA |
| (-260~-266) |  |  |
| Primer4 | TGAGTCCCTGCTATGTTGTCA | GCATACCCCGCCCAGAATTA |
| (-374~-368) |  |  |
| Primer5 | GTCTCAAACCCCTGACCTCA | CACCTGAAGTTGGACCTTAACA |
| (-1354~-1348) |  |  |
