# Supplementary material for: Aberrant ASPM expression mediated by transcriptional regulation of FoxM1 promotes the progression of gliomas
Source: J Cell Mol Med. 2020 Jul 15;24(17):9613–26. doi: 10.1111/jcmm.15435 (PMC7520292; doi:10.1111/jcmm.15435)
Supplement: Supplementary file 5 — Table S3 [file JCMM-24-9613-s005.docx]

**Supplementary Table 3. GO and KEGG Pathway enrichment analysis of differentially expressed genes**

| Category | Term | Count | % | *P* Value | Genes |
| --- | --- | --- | --- | --- | --- |
| GOTERM_BP_FAT | GO:0021987~cerebral cortex development | 4 | 0.0388425 | 2.75E-04 | KIF14, NTRK2, TACC3, ASPM |
| GOTERM_BP_FAT | GO:0000281~mitotic cytokinesis | 3 | 0.0291319 | 0.0026209 | KIF23, NUSAP1, KIF20A |
| GOTERM_BP_FAT | GO:0051301~cell division | 3 | 0.0291319 | 0.0121914 | KIF14, CKS2, BIRC5 |
| GOTERM_BP_FAT | GO:0000727~double-strand break repair via break-induced replication | 2 | 0.0194212 | 0.0145311 | GINS2, CDC45 |
| GOTERM_BP_FAT | GO:0044772~mitotic cell cycle phase transition | 2 | 0.0194212 | 0.0145311 | CDK1, CKS2 |
| GOTERM_BP_FAT | GO:0007018~microtubule-based movement | 3 | 0.0291319 | 0.0214691 | KIF14, KIF23, KIF20A |
| GOTERM_BP_FAT | GO:0019985~translesion synthesis | 2 | 0.0194212 | 0.0324049 | DTL, KIAA0101 |
| GOTERM_BP_FAT | GO:0007095~mitotic G2 DNA damage checkpoint | 2 | 0.0194212 | 0.0359415 | CDK1, FANCI |
| GOTERM_BP_FAT | GO:0034501~protein localization to kinetochore | 2 | 0.0194212 | 0.0359415 | CDK1, BUB1B |
| GOTERM_BP_FAT | GO:0007076~mitotic chromosome condensation | 2 | 0.0194212 | 0.0394654 | NCAPG, NUSAP1 |
| GOTERM_BP_FAT | GO:0008631~intrinsic apoptotic signaling pathway | 2 | 0.0194212 | 0.0394654 | MELK, SOD2 |
| GOTERM_BP_FAT | GO:0007094~mitotic spindle assembly checkpoint | 2 | 0.0194212 | 0.0429767 | BUB1, BUB1B |
| GOTERM_CC_FAT | GO:0030496~midbody | 6 | 0.0582637 | 8.65E-06 | KIF14, KIF23, CDK1, BIRC5, ASPM, KIF20A |
| GOTERM_CC_FAT | GO:0000778~nuclear chromosome kinetochore | 3 | 0.0291319 | 1.21E-04 | CENPA, BUB1B, NDC80 |
| GOTERM_CC_FAT | GO:0005876~spindle microtubule | 4 | 0.0388425 | 1.89E-04 | CDK1, NUSAP1, BIRC5, AURKA |
| GOTERM_CC_FAT | GO:0000942~ nuclear chromosome outer kinetochore | 2 | 0.0194212 | 0.0105247 | BUB1, NDC80 |
| GOTERM_CC_FAT | GO:0005871~kinesin complex | 3 | 0.0291319 | 0.0118646 | KIF14, KIF23, KIF20A |
| GOTERM_CC_FAT | GO:0032133~chromosome passenger complex | 2 | 0.0194212 | 0.0174808 | BIRC5, AURKA |
| GOTERM_CC_FAT | GO:0031298~replication fork protection complex | 2 | 0.0194212 | 0.0209409 | GINS2, CDC45 |
| GOTERM_CC_FAT | GO:0005813~centrosome | 5 | 0.0485531 | 0.021883 | KIF23, CDK1, CDC45, NCAPG, DTL |
| GOTERM_CC_FAT | GO:0005737~cytoplasm | 16 | 0.15537 | 0.0261571 | CDK1, DTL, DLGAP5, BRIP1, NUSAP1, BIRC5, CDC20, TACC3, FANCI, CDKN2C, NCAPG, PPP1R1A, BUB1, CENPU, MELK, ASPM |
| GOTERM_CC_FAT | GO:0072687~meiotic spindle | 2 | 0.0194212 | 0.0278253 | AURKA, ASPM |
| GOTERM_CC_FAT | GO:0031616~spindle pole centrosome | 2 | 0.0194212 | 0.0312497 | DLGAP5, AURKA |
| KEGG_PATHWAY | cfa04110: Cell cycle | 7 | 0.0679744 | 8.56E-06 | CDK1, CDC45, CCNB2, CDKN2C, BUB1, BUB1B, CDC20 |
| KEGG_PATHWAY | cfa04115: p53 signaling pathway | 4 | 0.0388425 | 0.0023209 | CDK1, CCNB2, RRM2, GTSE1 |
| KEGG_PATHWAY | cfa04914: Progesterone-mediated oocyte maturation | 4 | 0.0388425 | 0.0051321 | CDK1, CCNB2, BUB1, MAPK10 |
| KEGG_PATHWAY | cfa04512: ECM-receptor interaction | 4 | 0.0388425 | 0.0053008 | HSPG2, COL1A2, LAMC1, HMMR |
| KEGG_PATHWAY | cfa04114: Oocyte meiosis | 4 | 0.0388425 | 0.0093949 | CDK1, BUB1, CDC20, AURKA |
